# Sleeping Sickness Elimination in Sight: Time to Celebrate and Reflect, but Not Relax

**DOI:** 10.1371/journal.pntd.0001008

**Published:** 2011-02-22

**Authors:** Serap Aksoy

**Affiliations:** Yale School of Public Health, New Haven, Connecticut, United States of America

Sleeping sickness, also known as human African trypanosomiasis (HAT), is one of the neglected tropical diseases of sub-Saharan Africa that has plagued human health and agricultural development. In West Africa, infection with *Trypanosoma brucei gambiense* gives rise to a chronic disease that mainly affects humans. Infection with *T. b. gambiense* accounts for more than 90% of reported sleeping sickness cases. In East Africa, infection with *Trypanosoma brucei rhodesiense* generates acute disease in humans, and also circulates in a relatively unaffected livestock reservoir. Both Gambian and Rhodesian sleeping sickness are fatal if left untreated. The parasites are transmitted to the mammalian host through the bite of an infected tsetse fly.

Since its discovery a century ago, several waves of HAT epidemics have plagued the continent. During the colonial regimes, it was possible to bring about a steady decline in the number of reported *gambiense* cases from the 1930s onwards with systematic screening, treatment, and patient follow-up in western and central Africa. However, during the post-independence period of the 1960s when HAT cases declined, control programs within the endemic countries gradually were run down, resulting in a steep rise in incidence during the following 40 years. It has been difficult to estimate the true burden of HAT, as the disease affects the most neglected populations living in remote and rural settings where the majority of people affected are beyond the reach of health care systems and are not reported in any of the health metrics [1]. The World Health Organization (WHO) Expert Committee on HAT control and surveillance estimated in 1995 that the true number of cases was at least 10 times more than that reported considering the huge uncertainties between the reported cases and the factual field situation. Thus, from the 30,000 reported cases annually, it was estimated that some 300,000 infected individuals remained infected in the field [2].

In this issue of *PLoS Neglected Tropical Diseases*, Pere P. Simarro and colleagues from WHO report that the number of new cases diagnosed with HAT in 2009 has dropped below 10,000 for the first time in 50 years, signaling a possible end to the latest epidemic cycle as a major public health problem. This decline was achieved through an ambitious campaign led by WHO, and many nongovernmental organizations (NGOs) and thanks to a public–private partnership with Sanofi-Aventis and Bayer to donate and distribute the necessary drugs to WHO for use in affected countries. Without this generosity, patients would have no access to life-saving drugs, however unsatisfactory, given that many national health budgets are already stretched. A new nifurtimox/eflornithine combination treatment (NECT) was also developed recently that reduced both the cost of drugs and their delivery [3], [4]. Also crucial in HAT control was the recognition of the problem by African heads of state and governments during the African Union Summit in Lomé in 2000 where the Pan African Tsetse and Trypanosomiasis Eradication Campaign (PATTEC) was initiated with the objective to render Africa a tsetse- and trypanosomiasis-free continent [5]. While the news of reaching a probable elimination phase for HAT is most welcoming, sustainable management of disease control now poses a formidable challenge to endemic countries and health ministries that have to juggle a multitude of diseases when faced with shortages of funds. If the decline in the reported HAT cases is taken at face value, it could signal African governments to abandon their local control efforts and for funding agencies to modify their disease research priorities. Should this happen, it is most certain that epidemics will likely flare up in the near future as has happened in the past.

Given that HAT epidemiology varies significantly for *rhodesiense* and *gambiense* disease, the measures adopted will have to vary in different epidemiological settings. Nevertheless, since the distribution of sleeping sickness is limited to ancient and in many cases well-recognized foci, vigilant monitoring through surveillance at these foci should be continued for both forms of the disease. Towards this end, in February 2008 WHO launched the initiative of the Atlas of HAT to map all reported cases for the period 2000–2009 at the village level, which should be continued and updated [6]. Many reports have highlighted the feared merger of the *gambiense* and *rhodesiense* disease belts in Uganda, which would cause havoc given the different diagnostics and treatment regiments required for two diseases. Continued monitoring of diseases emerging in the potential merger zone has to remain a high priority for Uganda. For the *rhodesiense* disease with documented animal reservoirs, stringent implementation of animal treatments at livestock markets should continue to be a priority, especially in Uganda where cattle movements have been suggested to result in the continued spread of *rhodesiense* disease into new territories [7].

Despite intensive research into the biology of the trypanosomes and tsetse, to date the toolbox for diagnostics and treatment of sleeping sickness has remained extremely small and plagued with difficulties [8]. This is largely due to the complexity of the parasite's biology where an antigenic variation mechanism has hampered the development of mammalian vaccines. There are no prophylactic drugs. Furthermore, clinical tests applicable in the field for staging of disease have been difficult to develop. However, the basic knowledge accumulated on parasite and tsetse biology and the unprecedented technological advancements we are witnessing in science at this time provide the impetus for continued future research where the prospects for translational science are excellent. You can find a sample of the research that has been published in *PLoS Neglected Tropical Diseases* highlighting such promising discoveries in the special collection presented in this issue. It is most likely that innovative and interdisciplinary cutting-edge research will lead the way for improved and effective tools to monitor and prevent the next epidemic.

One promising area is in the development of DNA-based diagnostic tools such as the use of the loop-mediated isothermal amplification (LAMP) method for rapid detection of parasites in the field setting [9], [10]. This approach can also be applied to detect the circulating infectious parasites in natural tsetse populations or in reservoir animals to monitor the potential risk for human disease emergence during endemic periods. It has also been suggested that piggybacking HAT diagnostic efforts on the platforms already used for other diseases, such as tuberculosis as well as malaria and HIV, can lead to more sustainable surveillance efforts [11]. One immediate application is the use of a light-emitting diode (LED)–based fluorescence microscope developed for tuberculosis, which has been found to be highly sensitive for trypanosome diagnostics. Use of mini anion exchange centrifugation technique (mAECT) has improved diagnosis of *T. b. gambiense* infections, which are hard to detect due to low parasite densities in patient blood [12]. Continued research into safe, effective, and easy-to-use treatments are essential such as the recently rediscovered compound fexinidazole, which has entered into clinical development for the treatment of sleeping sickness [13].

A most effective means of preventing disease transmission is to remove tsetse flies, either at a local level (e.g., a group of villages) or regionally (covering large parts of a country or region). However, a major problem has been the cost and logistical difficulty of implementing such control programs. New research into variations of target design now indicate that catch efficiencies of the major human disease vector species *Glossina fuscipes fuscipes* can be improved by at least 10-fold, resulting in a considerable (more than 6-fold) cost saving for control programs [14]. Tsetse population genetics information has repeatedly identified extensive structuring in the field [15], [16]. Closer communication between the scientific community and vector control programs should be encouraged, as vector genetics information can benefit the ongoing control activities in the field and improve the sustainability of tsetse reduction efforts [17]. Eco-epidemiology is another growing discipline that can benefit HAT control efforts through the identification of potential disease loci and tsetse breeding sites for targeted control efforts as well as any potential impact of the anticipated climate change on disease patterns.

Finally, the genomes of the human host and the infectious trypanosomes have been sequenced [18]. Technological advancements have made it possible to determine the transcriptome of the trypanosome parasite at a single nucleotide level [19], opening up prospects for future studies where discoveries into disease stage–specific transcriptomes or proteomes will surely identify novel molecules for diagnostics and therapies. An international consortium of scientists brought together by WHO/TDR has recently announced that the tsetse vector genome (*Glossina morsitans morsitans*) is in the final stages of annotation [20], [21]. Furthermore, the United States National Institutes of Health has recently approved a comprehensive project to obtain the genome sequence of five additional tsetse species along with extensive transcriptome analysis. An immediate application of genomics discoveries could be on vector olfactory biology and could speed up investigations for the development of species-specific and cost-effective chemicals such as attractants to lure tsetse to targets and traps to reduce population densities [22], [23]. Lack of such chemicals for human disease–transmitting tsetse species has discouraged extensive use of vector control. Tsetse–trypanosome interactions can also now be investigated at the molecular level, and this has the potential to lead to pathways or targets to block the parasite's transmission ability in the vector.

In conclusion, it is time to celebrate the efforts of the HAT community that has succeeded in curbing the latest epidemic. However, it is important to remember that there are still 10,000 cases out there that need our attention. It is also important to remember that countries must put in place mechanisms and health personnel who can recognize and report any potential HAT cases in endemic areas to prevent the reemergence of disease. Finally, this is not the time to abandon research on tsetse and trypanosomes, but rather to exploit the accumulating knowledge in light of the technological advancements and build the toolbox by moving discoveries from bench to field for more effective diagnostics, therapies, and vector control methods.
